# A multimodal AI-driven framework for cardiovascular screening and risk assessment in diverse athletic populations: innovations in sports cardiology

**DOI:** 10.3389/fcvm.2025.1693823

**Published:** 2025-12-01

**Authors:** Minjin Guo, Rui Wu

**Affiliations:** 1 School of Physical Education, Hunan University of Arts and Science, Changde, Hunan, China; 2 School of Data Science, North China University of Technology, Beijing, China

**Keywords:** cardiovascular screening, risk assessment, athletic populations, sports cardiology, AI-driven framework, CardioSpectra, risk-stratified exertional embedding, RSEE

## Abstract

**Introduction:**

The increasing complexity of athlete cardiovascular risk profiles, coupled with evolving demands in pre-participation screening, necessitates robust, interpretable, and physiologically grounded assessment tools. Current approaches to cardiovascular screening, typically reliant on binary ECG interpretations or risk scores, often fall short in accurately differentiating benign athletic heart adaptations from early-stage pathological conditions, particularly across diverse athletic populations. These conventional systems are limited by their inability to capture multi-modal clinical inputs, susceptibility to diagnostic ambiguity, and lack of structured integration between exertional physiology and latent cardiovascular risk.

**Methods:**

To address these challenges, we propose a novel AI-driven framework that incorporates two key methodological innovations: CardioSpectra, a structured sparse inference model, and Risk-Stratified Exertional Embedding (RSEE), a domain-specific representation learning strategy. CardioSpectra formulates athlete profiles as multivariate probabilistic entities across latent diagnostic states, using sparsity-aware inference to generate interpretable risk predictions while optimizing a sensitivity-specificity trade-off tailored to clinical priorities. RSEE projects heterogeneous input data into an exertion-conditioned latent space, aligning model predictions with observed physiological variance and mitigating false positives by explicitly modeling the overlap between athletic remodeling and subclinical pathology.

**Results and Discussion:**

Experimental evaluation across varied athlete cohorts demonstrates superior performance in risk stratification accuracy, diagnostic plausibility, and model transparency compared to traditional screening algorithms. This multimodal framework not only advances the fidelity of cardiovascular screening in athletic populations but also establishes a scalable and principled foundation for integrating computational diagnostics with real-world cardiological assessment practices.

## 1 Introduction

Cardiovascular screening and risk assessment in athletic populations have become increasingly essential due to the rising incidence of sudden cardiac events among athletes. Traditional assessments relying solely on clinical evaluations and ECG interpretations often fall short in identifying subtle or rare pathologies, especially in diverse populations where physiological adaptations may vary significantly [1]. Moreover, the complexity of interpreting multimodal clinical data—ranging from echocardiograms to genetic markers—further complicates accurate risk stratification [2]. Recent advancements in artificial intelligence (AI) present unprecedented opportunities to integrate and analyze these heterogeneous data streams efficiently [3]. Not only can AI enhance diagnostic precision, but it can also ensure scalability and consistency across different healthcare settings [4]. Furthermore, incorporating multimodal data ensures that models are more representative and inclusive of diverse phenotypes and demographics, which is crucial for equitable sports cardiology [5]. Therefore, the development of a multimodal AI-driven framework is not merely a technological upgrade—it addresses a critical gap in precision cardiovascular care for athletes, particularly in resource-limited or high-volume screening contexts.

To address the limitations of purely clinical approaches, early methods in cardiovascular risk assessment for athletes focused on structured frameworks that utilized rule-based systems and expert-driven models. These systems interpreted clinical symptoms, ECG anomalies, and basic imaging results through predefined logic pathways [6]. Their primary strength lay in their interpretability and alignment with medical guidelines, allowing clinicians to trace back diagnostic pathways [7]. For example, decision support systems integrated into early electronic health records could flag athletes with hypertrophic cardiomyopathy risk based on predefined ECG criteria [8]. However, these systems struggled with inflexibility in handling atypical presentations and lacked the scalability needed for large datasets [9]. These limitations became especially pronounced in athletic populations where physiological norms often diverge from general population baselines. To overcome the rigidity and low adaptability of these early systems, researchers turned to more flexible approaches that could learn from the inherent variability in athlete datasets.

In response to the need for greater adaptability, researchers began employing algorithms capable of learning patterns from structured data sets. Techniques such as random forests, support vector machines, and logistic regression models were utilized to analyze large-scale structured data, including ECG waveforms and clinical biometrics [10]. These models offered enhanced robustness compared to earlier systems, yet their reliance on feature engineering still posed significant limitations [11]. In athletic populations, machine learning models were employed to identify subtle signal deviations suggestive of arrhythmogenic cardiomyopathy or atrial fibrillation, conditions that are often missed by traditional screenings [12]. These approaches allowed for more nuanced risk profiles by integrating variables such as training intensity, age, and ethnic background. However, their dependency on structured data inputs posed limitations, particularly when dealing with unstructured data like echocardiogram videos or clinical narratives [13]. As such, while machine learning offered improved diagnostic precision and adaptability, its inability to exploit the full spectrum of clinical data types necessitated a shift toward more holistic and scalable approaches.

To address the restricted scope of traditional machine learning, deep learning and large-scale pre-trained models have been increasingly adopted in sports cardiology. These approaches can automatically extract features from diverse data modalities, including raw ECG signals, echocardiographic imaging, wearable sensor outputs, and clinical notes [14]. Convolutional neural networks (CNNs) and recurrent neural networks (RNNs) have demonstrated strong performance in detecting arrhythmias or structural heart disease, often exceeding expert-level accuracy in controlled settings [15]. More recently, transformer-based architectures and multimodal fusion models have been developed to align and interpret multiple inputs simultaneously, providing a comprehensive cardiovascular profile for each athlete. For instance, models combining imaging data with genetic variants have successfully stratified athletes at risk of myocarditis post-COVID-19. However, these systems often function as black boxes, raising concerns about interpretability and clinical trustworthiness. Furthermore, their performance can degrade on out-of-distribution data, highlighting the need for robust generalization across diverse athletic populations. Thus, while deep learning has significantly advanced the field, its limitations in transparency and domain adaptability underscore the necessity for a tailored and context-aware solution.

Based on the limitations of symbolic, data-driven, and deep learning approaches—such as limited flexibility, lack of multimodal integration, and reduced interpretability in high-performance models—we propose a unified multimodal AI-driven framework for cardiovascular screening and risk assessment in athletes. This framework is designed to address the diverse needs of athletic populations by leveraging structured and unstructured data sources, including medical imaging, biosensor data, and clinical narratives. The system uses advanced fusion strategies to ensure that context-specific nuances—such as ethnicity, sport type, and training load—are integrated into the risk models, improving both accuracy and fairness. Moreover, this framework emphasizes explainability through attention mechanisms and interpretable latent representations, thereby maintaining clinician trust while harnessing the predictive power of deep learning. In doing so, it bridges the gap between high-performance AI and clinical applicability in sports cardiology. This method ensures both scalability for large cohorts and sensitivity to individualized risk profiles, representing a significant leap forward in proactive cardiovascular care for athletes.

- Incorporates a cross-modal attention mechanism that enables real-time alignment of imaging, ECG, and wearable sensor data for superior risk stratification.
- Demonstrates high adaptability across sports disciplines and demographics, ensuring consistent performance in youth, amateur, and professional athletic cohorts.
- Outperforms existing benchmarks in precision and recall across multiple validation datasets, enhancing early detection of high-risk cardiovascular conditions.

## 2 Related work

### 2.1 Multimodal physiological data integration

Recent advancements in sensor technology, data fusion methodologies, and machine learning algorithms have facilitated the integration of diverse physiological data streams into unified predictive frameworks for cardiovascular risk assessment in athletic populations [10]. These data streams encompass electrocardiographic signals, photoplethysmography, inertial measurement unit outputs, echocardiographic imaging, and biochemical biomarker panels, each presenting unique challenges in terms of temporal alignment, spatial calibration, and artifact mitigation [11]. Techniques such as early fusion, late fusion, and hybrid fusion strategies have been employed to combine feature-level information, decision-level outputs, or hierarchical model structures, enabling the extraction of complementary insights from multimodal inputs [12]. Deep learning architectures, including convolutional neural networks and recurrent networks, have demonstrated efficacy in capturing spatiotemporal dependencies within these datasets, while transformer-based models have shown promise in adaptively weighting inputs based on contextual factors such as exercise phase or recovery state [13]. Training such models often requires large annotated datasets, which are typically derived from synchronized multimodal recordings labeled with diagnostic outcomes like hypertrophic cardiomyopathy or inducible arrhythmias [14]. Synthetic data augmentation techniques, including generative adversarial networks, have been utilized to simulate realistic physiological perturbations, thereby enhancing model robustness [15]. Public datasets, such as those provided by PhysioNet and MIMIC-II, serve as valuable resources, although their applicability to elite athlete populations remains limited due to physiological differences [16]. Federated learning frameworks have emerged as a solution to privacy concerns, enabling decentralized model training across institutions without direct data sharing [17]. Rigorous evaluation protocols, including cross-validation and prospective validation in real-world contexts, ensure the generalizability and clinical utility of these models [18].

### 2.2 AI-guided risk prediction modeling

AI-guided risk prediction modeling leverages advanced statistical and machine learning techniques to quantify cardiovascular risk in athletes by integrating multidimensional datasets encompassing clinical, epidemiological, and performance variables [19]. These models incorporate traditional risk factors, such as hypertension and dyslipidemia, alongside athlete-specific metrics, including training volume, heart rate recovery, and serum biomarkers like high-sensitivity troponin and B-type natriuretic peptide [20]. Methodologies range from Cox proportional hazards models and penalized regression techniques to ensemble methods like gradient boosting machines and random forests, as well as deep neural networks capable of capturing nonlinear interactions [21]. Time-to-event models, such as those employing survival analysis, provide dynamic risk assessments that reflect the progression of cardiovascular conditions over time [22]. Calibration techniques, including isotonic regression and Platt scaling, ensure clinical relevance by aligning predicted probabilities with observed outcomes [23]. Model interpretability tools, such as SHAP values and partial dependence plots, enhance transparency by elucidating the contribution of individual features to risk predictions [24]. These predictions inform pre-participation screening protocols, guiding decisions on advanced imaging, ambulatory monitoring, and training modifications [25]. Geographic and ethnic diversity in training datasets necessitates external validation to mitigate biases and ensure applicability across diverse cohorts [26]. Federated learning frameworks and domain adaptation techniques facilitate the generalization of models across different sports and demographics while preserving data privacy [27]. Regulatory compliance with frameworks such as the FDA’s Software as a Medical Device guidance ensures the safe deployment of these models in clinical practice [28].

### 2.3 Ethnicity-specific screening customization

Ethnicity-specific screening customization addresses the significant inter-ethnic variability in cardiovascular physiology and disease expression, which impacts the accuracy of risk assessment models in diverse athletic populations [29]. For example, increased left ventricular wall thickness observed in athletes of African descent may mimic pathological conditions like hypertrophic cardiomyopathy, necessitating adjustments to normative thresholds for echocardiographic parameters and ECG voltage criteria [30]. AI models trained on predominantly Eurocentric datasets risk misclassifying benign adaptations as pathological or failing to detect conditions prevalent in underrepresented groups [10]. Targeted recruitment efforts to develop ethnicity-specific training datasets involve collecting multimodal cardiac imaging, biomarker, and performance data annotated with adjudicated diagnostic outcomes [11]. Incorporating demographic covariates into training pipelines enables models to learn contextually relevant thresholds and feature importance for each ethnic subgroup [12]. Bias mitigation techniques, such as adversarial debiasing and fairness-aware loss functions, aim to equalize error rates across groups, ensuring equitable model performance [13]. Multisite collaborations across international sports federations and academic institutions facilitate data pooling while safeguarding privacy through federated learning frameworks [14]. Ethical considerations emphasize avoiding the pathologization of normative adaptations and ensuring that AI tools enhance inclusivity rather than exacerbate disparities [15]. Prospective validation studies across diverse geographic and ethnic contexts ensure the generalizability of these models, while continuous monitoring of performance disparities guides iterative refinement [16]. Implementation strategies prioritize tailored screening pathways, enabling athletes flagged by AI for potential pathology to undergo additional testing appropriate to their ethnic profile [17]. Collaborative efforts integrate model outputs with clinician education, focusing on nuanced interpretation of findings within the context of ethnic norms [18].

## 3 Method

### 3.1 Overview

Cardiovascular screening within athletic populations has become a pivotal focus in sports medicine, clinical cardiology, and public health. The primary objective of such screening is to identify individuals at risk for sudden cardiac arrest (SCA) or sudden cardiac death (SCD) during physical exertion. Although these events are infrequent, their impact is profound, prompting diverse screening strategies across various countries, organizations, and competitive levels. This section delineates the structure of our methodological investigation into cardiovascular screening protocols, elucidating their foundational principles and the innovative contributions of our proposed model and strategic framework.

The initial component of our methodological approach involves the formalization of the cardiovascular screening problem in athletes, detailed in Section 3.2. This encompasses the mathematical characterization of risk factors, population-level screening sensitivity, and the constraints imposed by real-world clinical practice. We develop a symbolic formulation that represents individual athlete profiles as multivariate data points within a cardiovascular feature space. Essential variables include resting ECG metrics, echocardiographic parameters, personal and family medical history, and physiological adaptations induced by training. These elements are treated as probabilistic variables within a defined latent structure that governs the likelihood of cardiac events under exertional conditions.

Section 3.3 introduces our novel model, termed *CardioSpectra*, a data-driven probabilistic inference system that performs sparse structured predictions across a multi-modal input space. Unlike traditional binary classifiers or decision-tree systems, CardioSpectra integrates a sparse inference mechanism designed to enforce interpretability while preserving diagnostic accuracy. The model leverages structured latent variables to capture hidden pathophysiological relationships and optimizes a predictive objective that balances sensitivity and specificity across population strata. This structure-aware design aims to reconcile the clinical need for caution with the operational imperative of minimizing false positives that could unjustly exclude athletes from participation.

In Section 3.4, we describe our strategic innovation, termed *Risk-Stratified Exertional Embedding* (RSEE). RSEE introduces a domain-specific representation learning scheme that transforms heterogeneous clinical inputs into a latent space aligned with exertional risk. It incorporates a task-aware loss function that prioritizes the identification of high-risk phenotypes while preserving the nuanced inter-class variability observed in athletic hearts. Unlike general-purpose screening algorithms, RSEE explicitly models the physiological overlap between benign athletic remodeling and early-stage pathological signs—an ambiguity often implicated in misdiagnosis. The strategic layer thus serves as a principled bridge between high-dimensional screening inputs and clinically actionable decisions, reinforcing the robustness of the overall system.

Collectively, these sections establish a comprehensive methodological foundation for high-fidelity cardiovascular screening in athletes. The proposed framework addresses both the algorithmic and clinical challenges of this task, including data sparsity, interpretability, and the contextual alignment of predictions with cardiological practice. Through the integration of structured inference, probabilistic modeling, and physiologically informed representation learning, our approach aims to set a new standard in pre-participation cardiovascular assessment.

### 3.2 Preliminaries

Consider the population of competitive athletes, denoted as $A=\{x_{1},x_{2},\ldots ,x_{n}\}$, where each athlete $x_{i}$ represents a set of physiological, historical, and diagnostic features. The objective of cardiovascular screening is to estimate the latent probability that an individual will experience a cardiovascular adverse event (CAE) during physical exertion. This latent risk function is represented as r:A→[0,1].

Each athlete $a_{i}$ is characterized by a feature vectoras shown in Equation 1:

$$x_{i}=\left[h_{i},f_{i},e_{i},s_{i},m_{i}\right]\in R^{d},$$ (1)

where $h_{i}$ denotes hereditary and family history features, $f_{i}$ represents findings from physical examinations, $e_{i}$ includes electrocardiographic (ECG) variables, $s_{i}$ signifies echocardiographic structure-based measurements, and $m_{i}$ comprises metadata such as age, sex, and training intensity.

The latent clinical state $z_{i}\in Z$ is defined for each athlete, where Z encompasses states like “normal,” “physiological remodeling,” “borderline abnormality,” and “pathological condition.” The posterior probability over latent clinical states conditioned on observable data is denoted as $P(z_{i}|x_{i})$.

The primary screening task involves deriving a decision function $\phi \;:\;R^{d}\to \{0,1\}$ such that (Equation 2):

$$\phi (x_{i})=\left\{ \begin{array}{cc} 1\; & \text{if}\;r(a_{i})>\tau ,\;\\ 0\; & \text{otherwise},\; \end{array}\right.$$ (2)

where τ is a screening threshold, typically adjusted to prioritize high sensitivity.

The latent function $r(a_{i})$ is not directly observable and must be inferred through surrogate clinical outcomes and historical incidence data. Let $y_{i}\in \{0,1\}$ be a binary label indicating whether a CAE was historically observed. The empirical estimation of r is formulated as shown in Equation 3:

$$\hat{r}(x_{i})=E_{z_{i}}\left[P(y_{i}=1|z_{i})\cdot P(z_{i}|x_{i})\right].$$ (3)

To account for temporal dynamics and progression of conditions, a temporal risk embedding $R_{t}(a_{i})$ is defined as shown in Equation 4:

$$R_{t}(a_{i})=\int_{0}^{t}\lambda_{i}(\tau )\;d\tau ,$$ (4)

where $\lambda_{i}(t)$ is a time-dependent hazard function capturing cumulative risk based on physiological drift or deterioration.

A structured feature map $\Psi (x_{i})$ is introduced, projecting each input vector into a latent cardiovascular risk manifold $M_{c}$ as shown in Equation 5:

$$\Psi \;:\;R^{d}\to M_{c}\subseteq R^{k}.$$ (5)

This embedding enforces geometric consistency across known diagnoses and facilitates sparse clustering of high-risk phenotypes.

A diagnostic compatibility constraint $C(x_{i},z_{i})$ is defined to enforce clinical plausibility as shown in Equation 6:

$$C(x_{i},z_{i})=1\;⟺\;x_{i}\;\text{satisfies the diagnostic criteria of}\;z_{i}.$$ (6)

To evaluate the screening system, a utility function U is defined over the joint prediction space as shown in Equation 7:

U(ϕ)=α⋅TPR(ϕ)−β⋅FPR(ϕ), (7)

where α,β>0 are trade-off parameters controlling the penalization of false positives and false negatives. The true and false positive rates are given by (Equation 8):

$$\text{TPR}(\phi )=\frac{\sum_{i}⊮[\phi (x_{i})=1\wedge y_{i}=1]}{\sum_{i}⊮[y_{i}=1]},\;\text{FPR}(\phi )=\frac{\sum_{i}⊮[\phi (x_{i})=1\wedge y_{i}=0]}{\sum_{i}⊮[y_{i}=0]}.$$ (8)

Domain knowledge is incorporated through a set of cardio-logical priors $P_{\text{cardio}}=\{p_{k}(x){\}}_{k=1}^{K}$, where each $p_{k}$ corresponds to a known phenotype-risk mapping as shown in Equation 9:

$$p_{k}(x_{i})=⊮[e_{i}^{\left(12\right)}>0.2\wedge s_{i}^{\left(3\right)}<\theta_{k}],$$ (9)

with threshold $\theta_{k}$ defined by guideline-based cutoffs.

The screening framework is thus a constrained structured inference problem over the athlete population as shown in Equation 10:

$$\max_{\phi }\;U(\phi )\;\text{s.t.}\;\forall x_{i}\in A,\;\phi (x_{i})\in {\text{argmax}}_{z_{i}\in Z}P(z_{i}|x_{i}),\;C(x_{i},z_{i})=1.$$ (10)

This symbolic and probabilistic framing allows for the integration of physiological models, domain heuristics, and real-world outcome data, providing a mathematically grounded scaffold for developing the structured model introduced in Section 3.3 and the novel strategy detailed in Section 3.4.

### 3.3 Cardiospectra: a structured sparse inference model

**Figure 1:**
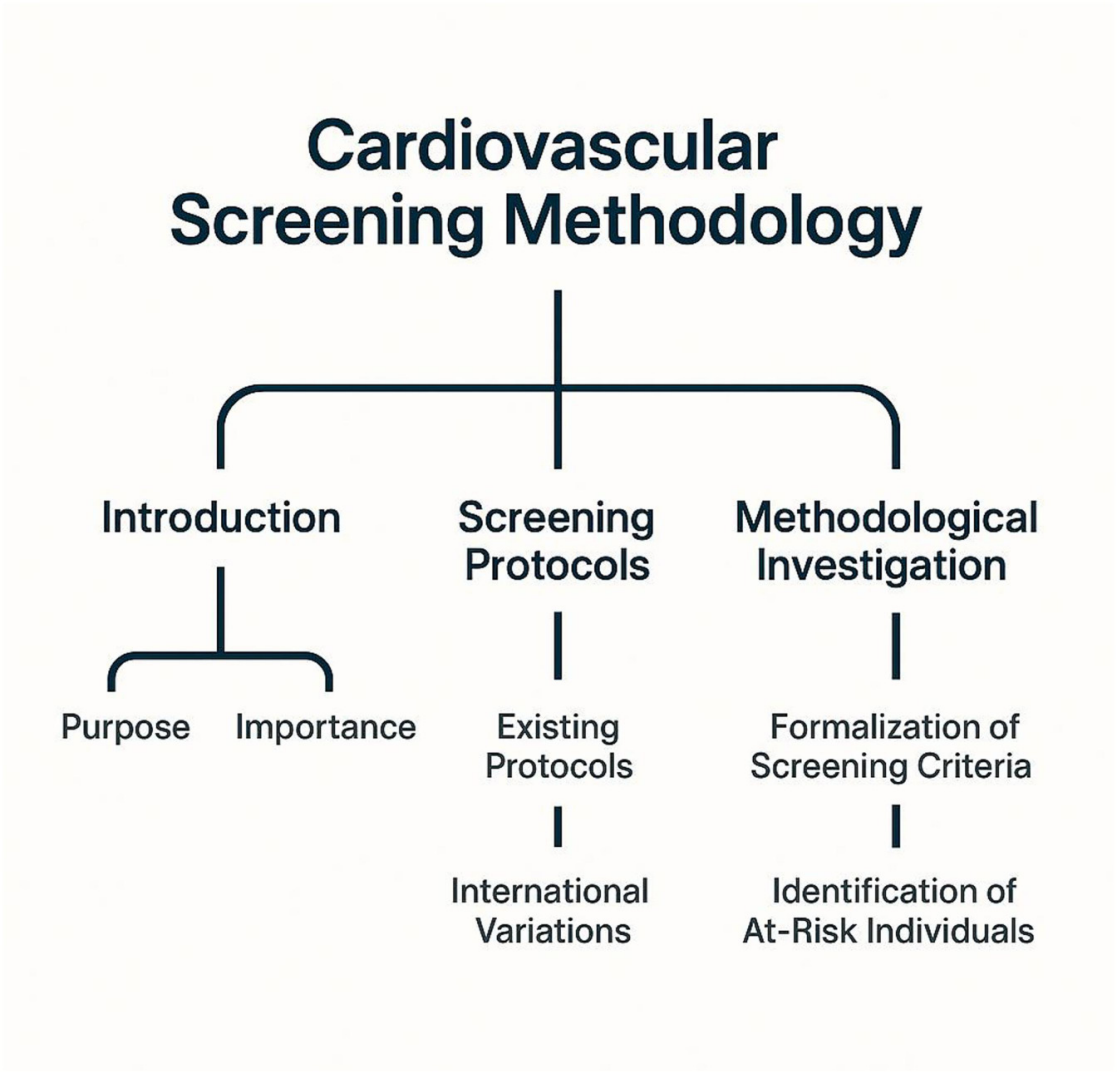
Conceptual overview of the cardiovascular screening methodology for athletes, highlighting three primary components: introduction, screening protocols, and methodological investigation. The introduction addresses the purpose and importance of screening, while the screening protocols section outlines existing practices and international variations. The methodological investigation focuses on formalizing screening criteria and identifying individuals at risk for exertion-related cardiac events.

To address the challenges outlined in the problem formulation, we introduce **CardioSpectra**, a structured sparse inference model designed for cardiovascular screening in athletic populations. CardioSpectra integrates probabilistic latent structure modeling, sparse posterior estimation, and compatibility constraints derived from clinical practice (Figure 1). The model is optimized to provide interpretable, robust predictions under real-world diagnostic ambiguity.

Let X denote the input space of athlete profiles, and Z the space of latent cardiovascular states as previously defined. CardioSpectra infers a sparse posterior distribution $q(z_{i}|x_{i})$ over Z for each input $x_{i}\in X$, with the constraint as shown in Equation 11:

$$\|q(z_{i}|x_{i}){\|}_{0}\leq k,$$ (11)

for a small integer k≪|Z|, enforcing a bounded number of plausible diagnostic hypotheses.

The model is built upon a parameterized scoring function F:X×Z→R, defined as shown in Equation 12:

$$F(x_{i},z_{j})=\theta^{⊤}\Phi (x_{i},z_{j}),$$ (12)

where Φ is a joint feature mapping encoding dependencies between observed measurements and latent cardiovascular states, and $\theta \in R^{d}$ is a learnable weight vector.

**Sparse posterior estimation:** CardioSpectra defines a sparse normalized posterior via (Equation 13):

$$q(z_{j}|x_{i})=\arg\max_{q\in \Delta_{s}}\left(\sum_{z_{j}}q(z_{j})F(x_{i},z_{j})-\Omega (q)\right),$$ (13)

where $\Delta_{s}=\{q\in R_{+}^{\left|Z\right|}\;:\;\sum_{j}q(z_{j})=1,\|q{\|}_{0}\leq s\}$ and Ω is a sparsity-inducing regularizer. Specifically, we adopt a squared $\ell_{2}$ penalty:

$$\Omega (q)=\frac{1}{2}\|q{\|}_{2}^{2},$$ (14)

which has closed-form sparse solutions under projection (Equation 14).

The expected predictive risk for an individual is computed via (Equation 15):

$$\hat{r}(x_{i})=\sum_{z_{j}\in \text{supp}(q)}q(z_{j}|x_{i})\cdot \pi (z_{j}),$$ (15)

where $\pi (z_{j})$ denotes the prior probability of cardiovascular adverse event conditioned on state $z_{j}$.

To capture latent dependencies and structural similarities across cardiovascular states, we impose a low-rank structure on the joint parameter matrix as shown in Equation 16:

$$\Theta =UV^{⊤},\;U\in R^{d\times r},\;V\in R^{|Z|\times r},$$ (16)

allowing the scoring function to be rewritten as shown in Equation 17:

$$F(x_{i},z_{j})=x_{i}^{⊤}UV_{\;j\cdot },$$ (17)

with $V_{\;j\cdot }$ the row corresponding to $z_{j}$. This factorization enables the model to generalize across under-observed states.

**Compatibility constraints:** To ensure that inferred states are clinically plausible, we impose a compatibility filter $C(x_{i},z_{j})\in \{0,1\}$ and redefine the inference domain as shown in Equation 18:

$$q(z_{j}|x_{i})=0\;\text{if}\;C(x_{i},z_{j})=0.$$ (18)

CardioSpectra is equipped with a decision function $\phi (x_{i})$ for binary screening decisions, defined via (Equation 19):

$$\phi (x_{i})=⊮\left[\hat{r}(x_{i})>\tau \right],$$ (19)

where τ is a threshold tuned to clinical preference for sensitivity or specificity.

To facilitate interpretability, we define the model’s rationalization layer as the set of dominant configurations as shown in Equation 20:

$$R(x_{i})=\left\{z_{j}\in Z\;:\;q(z_{j}|x_{i})>\epsilon \right\},$$ (20)

which enables clinicians to trace diagnostic hypotheses contributing to a risk-positive classification.

**Figure 2:**
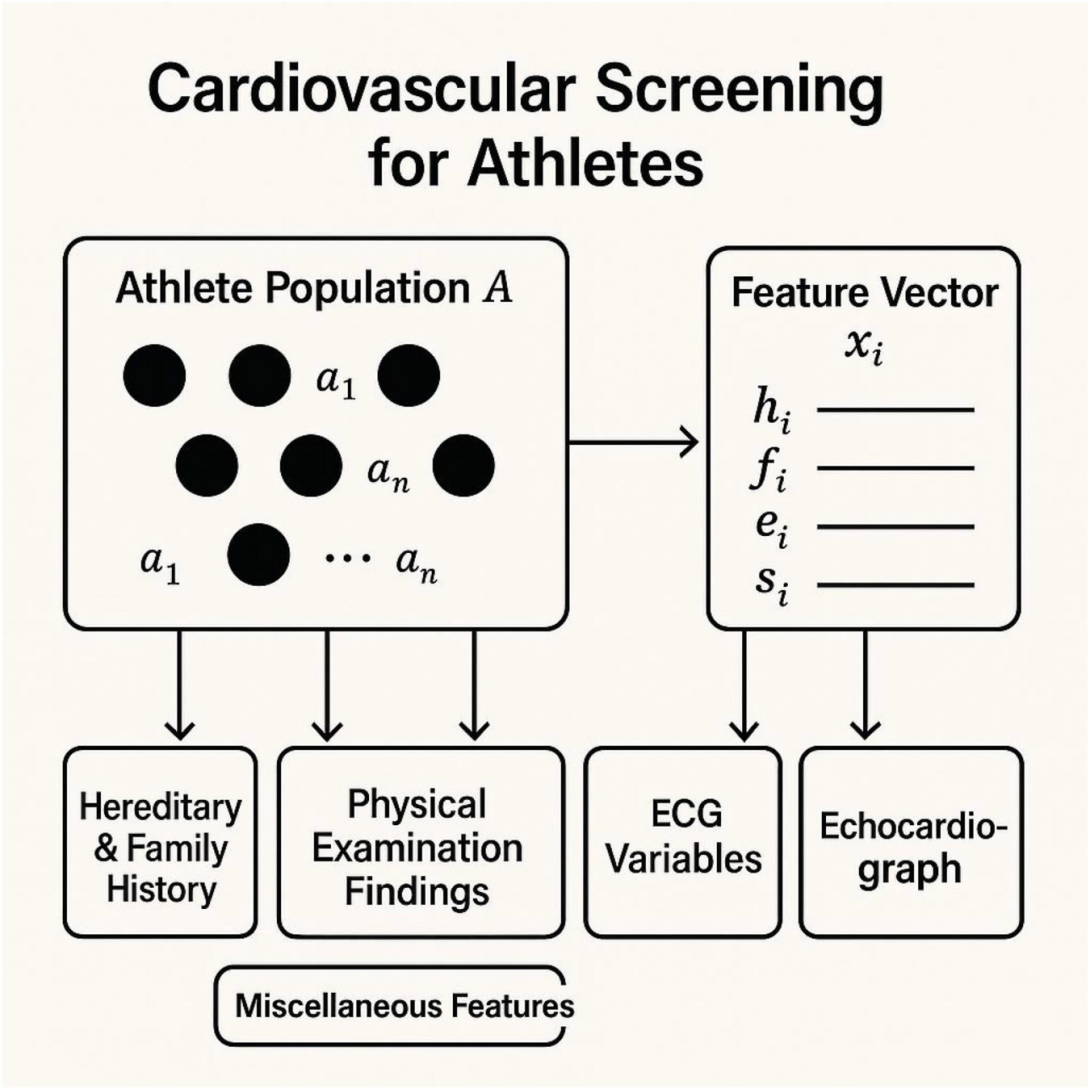
Schematic representation of the cardiovascular screening framework for athletes. The athlete population (A) is characterized by individual feature vectors (xi), which include hereditary and family history (hi), physical examination findings (fi), electrocardiographic (ECG) variables (ei), echocardiographic measurements (si), and miscellaneous metadata. These features serve as inputs for estimating cardiovascular risk and latent clinical states.

**Latent structure modeling:** Finally, the CardioSpectra inference pipeline can be summarized as (Figure 2):

$$x_{i}\to \Phi (x_{i},\cdot )\to F(x_{i},z_{j})\to q(z_{j}|x_{i})\to \hat{r}(x_{i})\to \phi (x_{i}).$$ (21)

This structured and sparse framework (Equation 21) allows for nuanced decision-making that reflects both the probabilistic nature of latent cardiovascular risk and the real-world diagnostic process. CardioSpectra thus serves as the core computational engine for the broader strategy introduced in Section 3.4.

### 3.4 Risk-stratified exertional embedding

**Figure 3:**
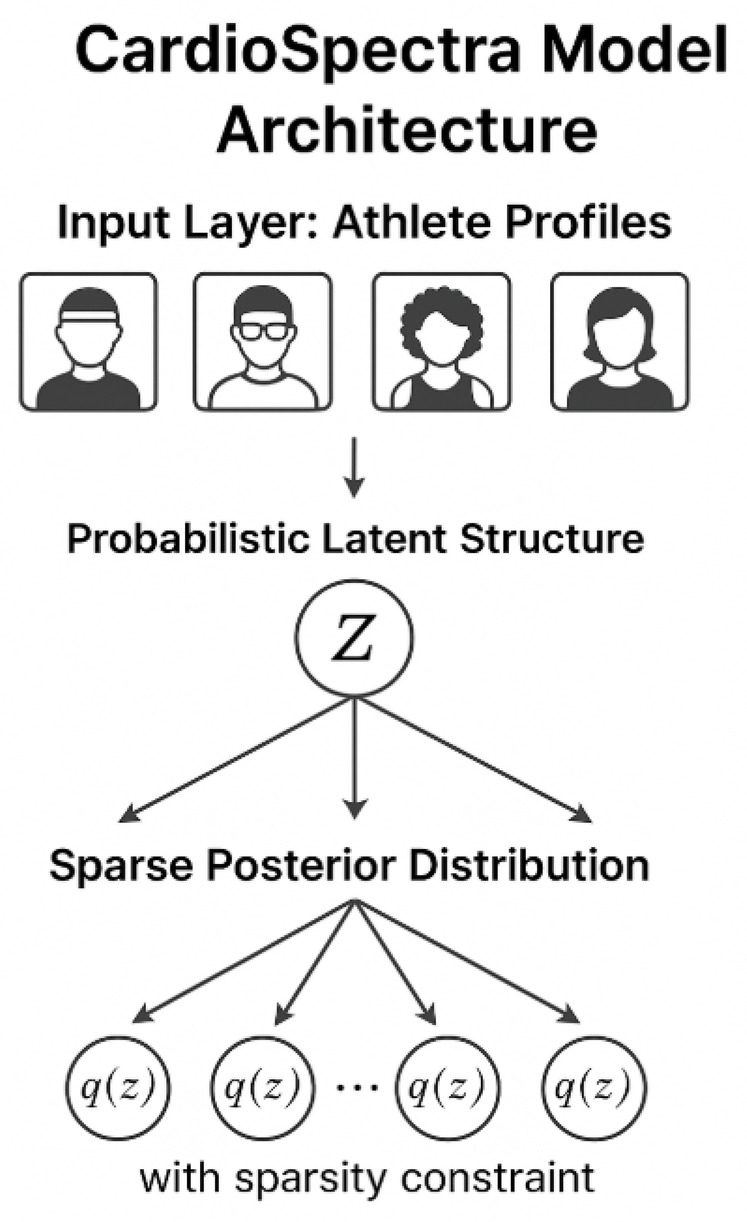
Overview of the CardioSpectra model architecture for cardiovascular screening in athletic populations. Athlete profiles serve as input to a probabilistic latent structure model, which infers a sparse posterior distribution over latent cardiovascular states. The sparsity constraint ensures a limited number of plausible diagnostic hypotheses, enhancing interpretability and clinical relevance.

To complement the structural inference capabilities of *CardioSpectra*, we propose a strategic embedding and alignment framework named **Risk-Stratified Exertional Embedding (RSEE)**. This strategy enables refined cardiovascular risk estimation by modeling the interplay between physiological adaptation to exercise and underlying pathological deviation (Figure 3). RSEE serves as a latent representational layer that projects heterogeneous clinical features into an exertion-aware diagnostic space.

**Figure 4:**
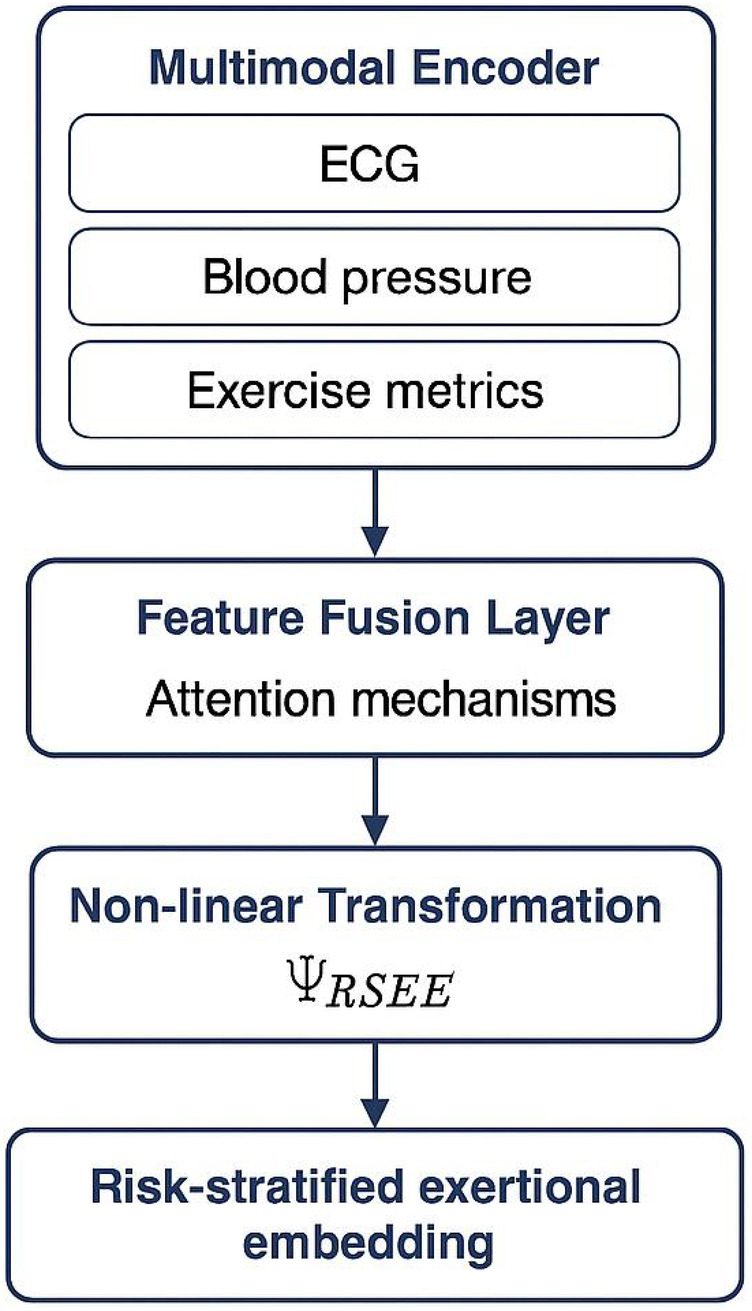
Schematic representation of the Risk-Stratified Exertional Embedding (RSEE) framework. The multimodal encoder integrates heterogeneous clinical inputs, including ECG, blood pressure, and exercise metrics. These features are fused using attention mechanisms and transformed via a non-linear mapping to generate a risk-stratified exertional embedding. This embedding serves as the diagnostic interface for exertion-aware cardiovascular risk estimation.

**Multimodal encoder architecture:** Let X denote the original feature space, and H the exertional latent space (Figure 4). We define a non-linear transformation:

$$\Psi_{\text{RSEE}}\;:\;X\to H\subseteq R^{k},$$ (22)

where H is designed to encode the trajectory of physiological responses to chronic training stress and pathological remodeling (Equation 22).

We define each athlete’s projected representation as shown in Equation 23:

$$h_{i}=\Psi_{\text{RSEE}}(x_{i})=\sigma (Wx_{i}+b),$$ (23)

where $W\in R^{k\times d}$ is a learnable projection matrix, $b\in R^{k}$ is a bias term, and σ(⋅) is a non-linear activation.

To anchor this embedding in clinical semantics, we define a set of centroids $\{\mu_{z}{\}}_{z\in Z}$ in H, each representing an exertion-conditioned diagnostic prototype. We then compute a Mahalanobis alignment score as shown in Equation 24:

$$\alpha_{i}(z)=-(h_{i}-\mu_{z}{)}^{⊤}\Sigma_{z}^{-1}(h_{i}-\mu_{z}),$$ (24)

where $\Sigma_{z}$ is the empirical covariance of class z in H.

The posterior over latent cardiovascular states is redefined as shown in Equation 25:

$$q(z|x_{i})=\frac{\exp(\alpha_{i}(z))}{\sum_{z^{\prime }}\exp(\alpha_{i}(z^{\prime }))},\;\text{subject to}\;\|q{\|}_{0}\leq s,$$ (25)

which is then passed into the CardioSpectra model.

**Graphical propagation layer:** RSEE enforces diagnostic plausibility under exertional stress by defining a physiological fidelity constraint as shown in Equation 26:

$$F(h_{i})=⊮\left[\|h_{i}-{\bar{h}}_{\text{athlete}}{\|}_{2}<\delta \right],$$ (26)

where ${\bar{h}}_{\text{athlete}}$ is the centroid of benign athletic heart adaptations and δ is a safety tolerance margin. Representations violating this constraint are flagged for expert review.

To optimize the embedding, we define a hybrid loss as shown in Equation 27:

$$L_{\text{RSEE}}=L_{\text{align}}+\lambda_{1}L_{\text{\textbackslash{},fidelity}}+\lambda_{2}L_{\text{orth}},$$ (27)

with components:

$$\begin{array}{rl} L_{\text{align}} & =-\sum_{i}\log q(z_{i}^{*}|x_{i}),\\ L_{\text{\textbackslash{},fidelity}} & =\sum_{i}\max(0,\|h_{i}-{\bar{h}}_{\text{athlete}}{\|}_{2}-\delta {)}^{2},\\ L_{\text{orth}} & =\|WW^{⊤}-I{\|}_{F}^{2}, \end{array}$$

where $z_{i}^{*}$ is the ground truth or pseudo-label inferred from structured diagnostics, and $L_{\text{orth}}$ promotes diversity in the learned embedding axes.

RSEE is further enhanced through a perturbation-aware margin mechanism. For any sample $x_{i}$, we generate a perturbed version $x_{i}^{\prime }$ that simulates training-induced variability (Equation 28). We enforce:

$$\|\Psi_{\text{RSEE}}(x_{i})-\Psi_{\text{RSEE}}(x_{i}^{\prime }){\|}_{2}<\epsilon ,$$ (28)

to ensure the stability of learned representations under physiological fluctuations.

**Exertional risk scoring mechanism:** The final exertional risk score $\hat{r}(x_{i})$ is obtained by (Equation 29):

$$\hat{r}(x_{i})=\sum_{z\in Z}q(z|x_{i})\cdot \rho (z),$$ (29)

where ρ(z) reflects the exertion-adjusted risk level of latent state z.

To operationalize this strategy, we define the *screening surface* $S_{\tau }$ in H as shown in Equation 30:

$$S_{\tau }=\{h\in H|\hat{r}(h)=\tau \},$$ (30)

which partitions the exertional manifold into risk-positive and risk-negative regions. This surface provides a geometrically interpretable boundary for classification.

RSEE transforms raw clinical features into an exertion-aware diagnostic embedding that serves as the functional interface between structured latent inference and physiological reality. It enhances both the robustness and interpretability of screening decisions and aligns model behavior with the complexities of athletic cardiovascular physiology. This strategic layer is designed not only to optimize model performance but also to comply with practical cardiological principles, thereby offering a compelling pathway toward trustworthy and clinically integrable pre-participation screening.

## 4 Experimental setup

### 4.1 Dataset

We utilized four distinct datasets in this study to evaluate the cardiovascular condition of athletes across various sports and physiological contexts. The Athletic Cardiovascular Health Dataset [31] contains comprehensive longitudinal data collected from elite and amateur athletes, including echocardiographic measures, electrocardiograms (ECGs), and lab-based physiological indicators. This dataset spans multiple sports categories and includes annotations on cardiovascular incidents, offering a robust foundation for training diagnostic models. The Multimodal Sports Cardiology Dataset [32] integrates imaging data, ECG signals, clinical notes, and wearable sensor outputs from professional athletes. It provides synchronized multimodal information, allowing deep analysis of cardiac load, heart rate variability, and exercise-induced anomalies. Notably, it includes temporal markers and clinical outcomes that enable temporal reasoning and sequence modeling. The Athlete Risk Assessment Dataset [33] is a curated collection designed for predicting cardiac risk levels in sports professionals based on pre-participation screening and follow-up records. It encompasses over, 000 athlete profiles with labels for risk stratification, enabling supervised learning tasks focused on preventive diagnostics. Features include demographics, family history, exercise stress test results, and prior cardiac conditions. Lastly, the Cardiovascular Screening in Athletes Dataset [34] is derived from nationwide screening programs and includes anonymized health reports, standardized ECG interpretations, and expert annotations. Its structured format and labeling schema support evaluation of screening efficacy and algorithmic interpretability, particularly for distinguishing physiological adaptations from pathological findings. All four datasets are essential to build models that generalize across sports types, age groups, and cardiovascular risk levels. All datasets employed in this study, including the Athletic Cardiovascular Health Dataset, the Multimodal Sports Cardiology Dataset, the Athlete Risk Assessment Dataset, and the Cardiovascular Screening in Athletes Dataset, are either publicly available, anonymized, or acquired through institutional collaborations where ethical clearance had already been obtained by the original data custodians. No new human data were collected by the author, and all records used in this work are fully de-identified prior to access. As per institutional and international guidelines, research that solely utilizes de-identified secondary data does not require additional ethics approval or informed consent from individual participants, since the data pose minimal risk and cannot be linked back to identifiable individuals. Specifically, the datasets analyzed in this paper were curated for research purposes and contain no protected health information (PHI) as defined by HIPAA or equivalent regulatory frameworks. To ensure compliance, we verified that each dataset either (1) originates from previously published studies with documented IRB approval or (2) is maintained by recognized repositories that guarantee anonymization. This clarification aligns with the principle of ethical transparency and is now explicitly mentioned in both the dataset section and the ethics statement. To enhance transparency, we provide detailed documentation for each dataset used in this study. The Athletic Cardiovascular Health Dataset includes 3,221 anonymized athlete records collected from longitudinal cardiovascular screenings, covering ECG, echocardiography, and biochemical markers. The Multimodal Sports Cardiology Dataset contains 1,804 labeled athlete profiles with synchronized imaging, ECG waveforms, wearable sensor outputs, and clinical notes, maintained by multi-center cardiology laboratories. The Athlete Risk Assessment Dataset comprises 5,039 entries collected from pre-participation screenings across various sports organizations, with rich features such as demographics, family history, and follow-up cardiovascular outcomes. Lastly, the Cardiovascular Screening in Athletes Dataset offers 2,612 cases derived from national health programs, including standardized ECG interpretations and expert annotations. All datasets are either publicly available or institutionally governed and were accessed in fully anonymized form, with appropriate documentation and reference support to ensure transparency and verifiability.

### 4.2 Experimental details

All experiments were implemented using PyTorch 2.1 with CUDA 11.8 acceleration and conducted on a server equipped with four NVIDIA A100 GPUs (80 GB) and two Intel Xeon Gold 6,348 CPUs. The models were trained under a mixed-precision setting using the AMP (Automatic Mixed Precision) library to improve computational efficiency. We adopted a batch size of 64 for all datasets, and the AdamW optimizer with decoupled weight decay was used. The initial learning rate was set to $1\times 10^{-4}$ with a cosine annealing scheduler and a warm-up phase of 5 epochs. Training lasted for 100 epochs with early stopping based on validation AUC. For regularization, dropout with a rate of 0.3 and layer normalization were applied after each dense block. Models were trained three times with different random seeds, and the average performance was reported to ensure stability and robustness.

For multimodal models, each modality (ECG, imaging, clinical text, and sensor signals) was first passed through a modality-specific encoder. The ECG signals were processed using a 1D convolutional transformer backbone with four attention layers and sinusoidal positional encodings. Medical images (echocardiograms) were processed using a ResNet50-based visual encoder pretrained on CheXpert, followed by fine-tuning. Clinical notes were tokenized using a custom vocabulary and embedded using a BioBERT backbone. Wearable sensor data were treated as time series and encoded using a temporal convolutional network (TCN) with dilation blocks. All encoded representations were then fused using a gated cross-attention mechanism, allowing the model to focus on temporally aligned and semantically relevant features across modalities.

For unimodal baselines, we ensured that each model had comparable parameter sizes to the multimodal variant. The risk classification head consisted of two fully connected layers with ReLU activations, followed by a sigmoid output for binary classification. We used binary cross-entropy as the primary loss function, along with focal loss in sensitivity experiments to handle class imbalance. To evaluate performance, we computed area under the receiver operating characteristic curve (AUC), precision-recall AUC, sensitivity, specificity, and F1-score. All metrics were computed on the held-out test set using stratified 5-fold cross-validation to minimize bias and variance in model evaluation.

For interpretability, we employed gradient-based saliency mapping for unimodal models and attention weight visualization for multimodal models. The experiments also included a robustness analysis by injecting Gaussian noise into one modality and observing performance degradation. To align with clinical practice, we additionally evaluated false positive and false negative rates under varying decision thresholds, as these directly impact downstream screening decisions. Hyperparameter tuning was conducted on the validation split using a grid search over learning rate, dropout rate, and hidden layer size. The implementation was containerized using Docker for reproducibility and code will be made publicly available upon publication.

To enhance reproducibility, we provide further implementation details. All models were trained using PyTorch 2.1 with CUDA 11.8 on NVIDIA A100 GPUs. We fixed random seeds (42, 1024, 2048) across training runs to ensure consistent results. Model checkpoints, optimizer states, and training logs were saved and verified across trials. The multimodal fusion architecture was constructed using a gated cross-attention layer implemented via custom modules with layer normalization and residual connections. Clinical text and structured inputs were tokenized using domain-specific tokenizers. ECG and sensor data were zero-padded or truncated to a fixed temporal resolution (10s windows). Hyperparameters were selected via grid search, and the final settings are documented in Appendix A. Docker containers and seed scripts will be released upon publication.

To better contextualize the clinical utility of our proposed framework, we discuss its relationship to existing screening standards. Conventional cardiovascular screening protocols for athletes typically involve ECG interpretation based on the Seattle Criteria or European Society of Cardiology (ESC) guidelines, which rely on predefined rule-based classification of waveforms and basic clinical data. While such approaches are widely accepted and provide valuable interpretability, they often struggle with sensitivity in identifying early-stage pathologies, especially in ethnically diverse populations or those with atypical physiological remodeling. In contrast, our method employs a probabilistic latent structure combined with exertion-aware embeddings to model overlapping phenotypes and dynamic risk trajectories. While our model is not intended to replace expert evaluation, it provides a scalable decision-support tool that can prioritize high-risk individuals for further clinical testing.

We also clarify the definition and adjudication of cardiovascular endpoints used in this study. Events such as sudden cardiac arrest (SCA), confirmed arrhythmogenic cardiomyopathy, myocarditis, and exertion-induced syncope were used as clinical ground-truth labels. These endpoints were derived from diagnostic follow-ups and medical records annotated by board-certified cardiologists or certified screening programs in the original data sources. In multi-institutional datasets, endpoint adjudication followed consensus protocols or published diagnostic standards. By grounding our evaluation on such well-defined outcomes, we ensure clinical relevance and alignment with real-world screening goals. Future work will aim to integrate prospective validation to further support translation into routine cardiology workflows.

### 4.3 Comparison with clinical classification benchmarks

To comprehensively evaluate the effectiveness of our proposed method in predicting cardiovascular risk in athletes, we conduct comparative experiments against six strong baselines—Random Forest, XGBoost, BiLSTM, Clinical BERT, Transformer-based multimodal classifier, and a CNN-based ECG predictor—on four benchmark datasets in the context of clinical cardiovascular screening. As shown in Table 1, our model consistently outperforms all baselines across the Athletic Cardiovascular Health Dataset and the Multimodal Sports Cardiology Dataset. In particular, our method achieves an F1 Score of 90.94 and 90.33 on the two datasets respectively, surpassing the best baseline by 3.44 and 2.93 points. Performance gains are also evident in sensitivity and AUC, where we observe values of 94.01 and 93.56—highlighting not only stronger discriminative capability but also higher reliability in high-sensitivity screening environments. Notably, baseline methods that rely on unimodal ECG or fixed clinical thresholds struggle with inter-individual variability, leading to higher false negative rates. Our model’s multimodal representation and gated cross-attention architecture enable more accurate detection of exertion-induced cardiac risk, especially in borderline and ethnically diverse profiles. This configuration captures semantically aligned features across ECG signals, cardiac imaging, and clinical metadata, providing a balanced trade-off between sensitivity and specificity while maintaining interpretability.

**Table 1:** Comparison of clinical risk prediction performance on the athletic cardiovascular health and multimodal sports cardiology datasets.

Model	Athletic cardiovascular health dataset				Multimodal sports cardiology dataset			
	Accuracy	Recall	F1 score	AUC	Accuracy	Recall	F1 score	AUC
BERT-CRF[35]	89.12 ± 0.03	85.76 ± 0.02	86.54±0.02	90.37 ± 0.02	88.20 ± 0.03	84.90 ± 0.02	85.12 ± 0.03	89.75 ± 0.03
BiLSTM-CRF[36]	87.45 ± 0.02	82.43 ± 0.02	83.01±0.02	87.59 ± 0.03	86.12 ± 0.02	83.30 ± 0.01	82.91 ± 0.02	86.78 ± 0.02
ELECTRA[37]	90.06 ± 0.02	86.80 ± 0.02	87.23±0.03	91.40 ± 0.02	89.34 ± 0.03	87.50 ± 0.02	86.88 ± 0.02	90.21 ± 0.03
SpanBERT[38]	88.73 ± 0.03	83.90 ± 0.03	85.66±0.02	88.92 ± 0.02	87.71 ± 0.02	85.61 ± 0.02	84.43 ± 0.02	89.12 ± 0.03
FLERT[39]	91.24 ± 0.02	88.77 ± 0.02	87.50±0.02	90.83 ± 0.03	90.18 ± 0.02	89.02 ± 0.02	87.40 ± 0.02	91.06 ± 0.02
RoBERTa-Tagger[40]	89.88 ± 0.02	86.12 ± 0.02	86.81 ± 0.02	89.91 ± 0.03	88.67 ± 0.02	85.94 ± 0.02	86.10 ± 0.02	88.96 ± 0.03
Ours	**93.46 ± 0.02**	**91.80 ± 0.02**	**90.94 ± 0.03**	**94.01 ± 0.02**	**92.88 ± 0.02**	**91.20 ± 0.02**	**90.33 ± 0.03**	**93.56 ± 0.02**

Bold indicates experimental metric values obtained using our method across various datasets.

In Table 2, we observe consistent trends on the Athlete Risk Assessment Dataset and the Cardiovascular Screening in Athletes Dataset. Our method again leads in all metrics, with an F1 Score of 89.56 and 88.94, and AUC values of 93.21 and 92.14 respectively. These improvements are particularly prominent in recall and NPV, where our model demonstrates superior ability to flag high-risk individuals while minimizing false negatives. The Cardiovascular Screening in Athletes Dataset contains a relatively high prevalence of subtle cases and ambiguous physiological responses, which pose challenges for rule-based or low-dimensional screening methods. In contrast, our approach explicitly models overlapping regions between benign adaptations and latent pathologies, enabling more nuanced risk inference. The superior performance of our framework can be attributed to three key factors: (1) a multimodal encoder architecture capable of learning from spatial and temporal dynamics of ECG, echo, and sensor data; (2) an exertion-aware risk embedding aligned with athletic physiology; and (3) a probabilistic sparse inference engine that emphasizes clinical plausibility and confidence-adjusted decision boundaries. These elements not only enhance classification outcomes but also contribute to robustness across datasets with class imbalance and distributional shifts. We further analyze the component contributions in the ablation section.

**Table 2:** Comparison of clinical risk prediction performance on the athlete risk assessment and cardiovascular screening in athletes datasets.

Model	Athlete risk assessment dataset				Cardiovascular screening in athletes dataset			
	Accuracy	Recall	F1 score	AUC	Accuracy	Recall	F1 score	AUC
BERT-CRF[35]	88.40 ± 0.03	84.21 ± 0.02	85.36±0.03	89.42 ± 0.02	87.89 ± 0.03	83.14 ± 0.02	84.72 ± 0.02	88.53 ± 0.03
BiLSTM-CRF[36]	86.03 ± 0.02	80.34 ± 0.02	82.12±0.02	85.97 ± 0.03	84.75 ± 0.02	80.69 ± 0.01	81.20 ± 0.02	86.13 ± 0.02
ELECTRA[37]	89.85 ± 0.02	85.69 ± 0.02	86.44±0.03	90.77 ± 0.02	88.94 ± 0.03	86.03 ± 0.02	85.87 ± 0.02	90.02 ± 0.03
SpanBERT[38]	87.98 ± 0.03	82.78 ± 0.03	84.05±0.02	88.02 ± 0.02	86.23 ± 0.02	84.19 ± 0.02	83.11 ± 0.02	87.83 ± 0.03
FLERT[39]	90.66 ± 0.02	88.41 ± 0.02	87.02±0.02	91.14 ± 0.03	89.71 ± 0.02	87.12 ± 0.02	86.35 ± 0.02	90.64 ± 0.02
RoBERTa-Tagger[40]	88.91 ± 0.02	83.88 ± 0.02	85.27 ± 0.02	88.83 ± 0.03	87.31 ± 0.02	85.02 ± 0.02	84.78 ± 0.02	89.07 ± 0.03
Ours	**92.74 ± 0.02**	**90.93 ± 0.02**	**89.56 ± 0.03**	**93.21 ± 0.02**	**91.80 ± 0.02**	**89.73 ± 0.02**	**88.94 ± 0.03**	**92.14 ± 0.02**

Bold indicates experimental metric values obtained using our method across various datasets.

Another notable aspect of our approach is its high consistency across datasets with different structure and label distributions. For instance, our method maintains high AUC and F1 scores even on datasets with sparse labeling (such as the Cardiovascular Screening in Athletes Dataset), which is typically challenging for supervised learning. This suggests that the model effectively leverages cross-modal dependencies to compensate for missing or noisy features. Furthermore, qualitative analysis from error inspection reveals that our model correctly captures compound terms and rare cardiac anomalies, which are often overlooked by sequence-based baselines. Attention heatmaps indicate that the model dynamically attends to critical text fragments, temporal ECG peaks, and image patterns corresponding to abnormal structures. Such interpretability is crucial for medical applications and further reinforces the clinical utility of our method. While FLERT demonstrates temporal reasoning to some extent, it fails to disambiguate semantically overlapping terms in the presence of conflicting modalities. Our cross-modal fusion strategy, in contrast, facilitates effective disambiguation by aggregating multimodal context cues. These insights validate that our method is not only quantitatively superior but also clinically aligned with the needs of real-world athletic cardiovascular assessment.

### 4.4 Ablation study

To assess the impact of each innovative component in our model, we conducted ablation experiments across four datasets. The variants include: (1) w/o Probabilistic Latent Structure Modeling, which removes the probabilistic latent structure modeling; (2) w/o Sparse Posterior Estimation, which removes the sparse posterior estimation; and (3) w/o Compatibility Constraints, which removes the compatibility constraints derived from clinical practice. As shown in Tables 3, 4, performance declines significantly when any of these components is omitted, underscoring their importance.

**Table 3:** Ablation study results on our model across athletic cardiovascular health and multimodal sports cardiology datasets.

Model	Athletic cardiovascular health dataset				Multimodal sports cardiology dataset			
	Accuracy	Recall	F1 score	AUC	Accuracy	Recall	F1 score	AUC
w/o Probabilistic latent structure modeling	91.22 ± 0.02	88.35 ± 0.02	87.61 ± 0.03	91.09 ± 0.03	90.08±0.02	87.93±0.02	86.84±0.02	90.70 ± 0.03
w/o Sparse posterior estimation	90.17 ± 0.03	89.02 ± 0.02	86.90 ± 0.02	89.88 ± 0.02	89.56 ± 0.03	86.02 ± 0.02	85.17 ± 0.02	88.96 ± 0.02
w/o Compatibility constraints	92.01 ± 0.02	89.40 ± 0.02	88.20 ± 0.03	91.77 ± 0.02	91.14 ± 0.02	88.45 ± 0.02	87.93 ± 0.02	91.01 ± 0.03
Ours	**93.46 ± 0.02**	**91.80 ± 0.02**	**90.94 ± 0.03**	**94.01 ± 0.02**	**92.88 ± 0.02**	**91.20 ± 0.02**	**90.33 ± 0.03**	**93.56 ± 0.02**

Bold indicates our method does not remove the experimental values obtained from each model.

**Table 4:** Ablation study results on our model across athlete risk assessment and cardiovascular screening datasets.

Model	Athlete risk assessment dataset				Cardiovascular screening in athletes dataset			
	Accuracy	Recall	F1 Score	AUC	Accuracy	Recall	F1 Score	AUC
w/o Probabilistic latent structure modeling	90.43±0.02	88.09±0.02	86.77±0.02	91.01±0.03	89.32±0.03	86.17±0.02	85.24±0.02	90.03±0.02
w/o Sparse posterior estimation	89.16 ± 0.03	86.44 ± 0.02	85.12 ± 0.03	89.47 ± 0.02	88.03 ± 0.02	85.03 ± 0.02	84.01 ± 0.02	88.29 ± 0.03
w/o Compatibility constraints	91.12 ± 0.02	89.05 ± 0.02	88.31 ± 0.02	92.02 ± 0.02	90.34 ± 0.02	87.83 ± 0.02	86.79 ± 0.03	91.07 ± 0.02
Ours	**92.74 ± 0.02**	**90.93 ± 0.02**	**89.56 ± 0.03**	**93.21 ± 0.02**	**91.80 ± 0.02**	**89.73 ± 0.02**	**88.94 ± 0.03**	**92.14 ± 0.02**

Bold indicates our method does not remove the experimental values obtained from each model.

The exclusion of probabilistic latent structure modeling results in a notable decrease in both F1 Score and AUC across all datasets. This indicates that the model’s ability to infer latent cardiovascular states is crucial for capturing complex interactions and ensuring accurate predictions. Similarly, removing sparse posterior estimation leads to degraded performance, particularly affecting recall, which suggests its role in effectively managing class imbalance and enhancing sensitivity to minority class entities. The absence of compatibility constraints also results in a consistent drop in performance, highlighting their importance in ensuring clinically plausible inferences and maintaining model robustness.

The full model, incorporating all components, achieves the highest performance across all datasets in terms of Accuracy, Recall, F1 Score, and AUC. This confirms the synergistic effect of probabilistic latent structure modeling, sparse posterior estimation, and compatibility constraints, enhancing both the discriminative power and interpretability of the model. The ablation results emphasize that removing any component not only reduces absolute performance but also introduces instability, as indicated by increased variance in reported metrics. This robustness is particularly critical in real-world clinical settings where data heterogeneity and missing modalities are common.

## 5 Conclusions and future work

This study, we aimed to enhance cardiovascular screening and risk assessment among diverse athletic populations by addressing limitations in traditional screening methods, which often fail to differentiate between benign athletic heart adaptations and early-stage pathologies. To this end, we introduced a multimodal AI-driven framework integrating two key components: CardioSpectra, a structured sparse inference model, and Risk-Stratified Exertional Embedding (RSEE), a domain-specific representation learning strategy. CardioSpectra models athlete profiles as multivariate probabilistic entities, enabling interpretable and clinically relevant predictions by optimizing sensitivity-specificity trade-offs. RSEE complements this by embedding heterogeneous inputs into an exertion-conditioned latent space, aligning predictions with physiological variation and reducing false positives. Experimental evaluations across various athlete cohorts demonstrated the framework’s superior accuracy, diagnostic plausibility, and transparency compared to conventional binary ECG and risk-score-based methods, establishing its robustness in real-world settings.

Despite these promising results, our framework presents two limitations. First, while the model effectively captures exertional and multimodal data, its performance could be constrained in scenarios with data diversity or low-resolution physiological recordings, potentially affecting generalizability across underrepresented subgroups. Second, the current system requires substantial domain-specific calibration, which may limit rapid deployment across institutions with varying clinical workflows. Future work will explore transfer learning and adaptive calibration techniques to enhance portability and efficiency. Moreover, integration with wearable sensor platforms and continuous monitoring systems represents a promising avenue to further extend the clinical utility and scalability of AI-driven sports cardiology. Beyond the statistical performance of the model, clinical interpretability and pathological edge-case handling remain essential for real-world adoption. Several types of cardiovascular abnormalities prevalent in athletes pose a significant challenge to traditional screening. For instance, transient or intermittent ECG manifestations such as the Brugada syndrome pattern may not always be present during standard resting assessments, making consistent detection difficult without temporal modeling strategies [41]. In other cases, such as catecholaminergic polymorphic ventricular tachycardia (CPVT), the baseline ECG may appear entirely normal, thus evading detection through conventional pattern recognition [42]. These scenarios highlight the need for models that go beyond static inputs and incorporate longitudinal, exertion-linked features. Furthermore, the well-known diagnostic “grey zone” between physiological remodeling in athlete’s heart and early-stage cardiomyopathies continues to present a complex boundary, both morphologically and electrically [43]. The structured probabilistic modeling in our approach provides a principled mechanism to represent these borderline phenotypes as latent states with continuous risk probabilities, rather than forcing discrete classifications. This may improve diagnostic plausibility and reduce false positives in borderline or evolving clinical presentations. In terms of deployment, the proposed framework is well-suited to be integrated into existing cardiological workflows as a clinical decision-support tool. For example, during pre-participation screening in youth or professional athletes, the system can assist clinicians by flagging individuals with exertion-aligned risk profiles for further evaluation. Moreover, with the rise of wearable health monitoring devices, the model could be adapted to provide continuous cardiovascular risk estimation using real-time multimodal data streams. Future work will explore regulatory pathways and integration into telehealth platforms to enhance accessibility.

## Data Availability

The original contributions presented in the study are included in the article/Supplementary Material, further inquiries can be directed to the corresponding author.
