# Supplementary figures and images for: A multimodal AI-driven framework for cardiovascular screening and risk assessment in diverse athletic populations: innovations in sports cardiology
Source: Front Cardiovasc Med. 2025 Dec 1;12:1693823. doi: 10.3389/fcvm.2025.1693823 (PMC12702950; doi:10.3389/fcvm.2025.1693823)

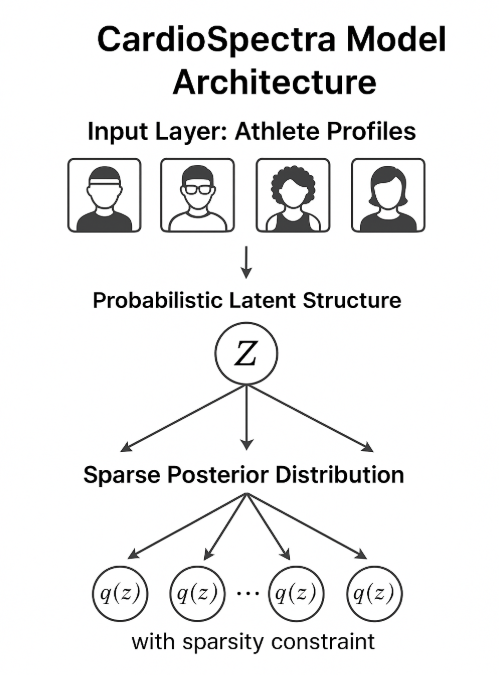

Supplement: Supplementary file 1 [file Image1.png]
